# Changes in microRNA expression profile in hippocampus during the acquisition and extinction of cocaine-induced conditioned place preference in rats

**DOI:** 10.1186/1423-0127-20-96

**Published:** 2013-12-20

**Authors:** Chun-Lin Chen, Hailin Liu, Xiaowei Guan

**Affiliations:** 1 Department of Human Anatomy, Nanjing Medical University, 140 Hanzhong Road, Nanjing 210029, China; 2 Department of Biological Science, National Sun Yat-Sen University, Kaohsiung 80424, Taiwan; 3 Department of Anesthesiology, Huai’an First People’s Hospital, Nanjing Medical University, Huaian 223300, China

**Keywords:** Cocaine, Hippocampus, microRNA, Microarray, Conditioned place preference

## Abstract

**Background:**

MicroRNA (miRNA) emerges as important player in drug abuse. Yet, their expression profile in neurological disorder of cocaine abuse has not been well characterized. Here, we explored the changes of miRNA expression in rat hippocampus following repeated cocaine exposure and subsequent abstinence from cocaine treatment.

**Results:**

Conditioned place preference (CPP) procedure was used to assess the acquisition and extinction of cocaine-seeking behavior in rats. MiRNA microarray was performed to examine miRNAs levels in rat hippocampus. Quantitative RT-PCR was conducted to further confirm results in microarray study. Finally, bioinformatic predictions were made to suggest potential target genes of cocaine-responsive miRNA in this study. MiRNA array found that 34 miRNA levels were changed in rat hippocampus while acquiring cocaine CPP and 42 miRNAs levels were altered after the cocaine-induced CPP were extinguished, as compared to normal controls. The findings from qRT-PCR study support results from microarray analysis.

**Conclusions:**

The current study demonstrated dynamic changes in miRNA expression in rat hippocampus during the acquisition and extinction of cocaine-induced CPP. Some miRNAs which have been previously reported to be involved in brain disorders and drug abuse, including miR-133b, miR-134, miR-181c, miR-191, miR-22, miR-26b, miR-382, miR-409-3p and miR-504, were found to be changed in their expression following repeated cocaine exposure and subsequent abstinence from cocaine treatment. These findings may extend our understanding of the regulatory network underlying cocaine abuse and may provide new targets for the future treatment of drug abuse.

## Background

Drug induces changes of gene expression in the brain, which are thought to contribute to drug addictive behaviors, such as loss of control over drug intake and seeking [1-3]. Lots of genes have been identified to play important roles in the development of cocaine addiction [4,5]. Notably, many of these genes exert their regulatory functions on cocaine abuse by forming a complex regulatory network. Yet, we still have not gained a full picture about this regulatory network, and the “master regulator” in the process of cocaine addiction remains to be identified.

MicroRNAs (miRNAs) are small, endogenous non-protein coding RNA molecules that regulate gene expression at the post-transcriptional level by interacting with target message RNAs (mRNAs) [6]. Recent studies have investigated the roles of miRNAs in biochemical, molecular and behavioral responses to cocaine. For example, miR-181, miR-124 and let-7d are suggested to be involved in cocaine-induced nervous plasticity and cocaine-induced conditioned place preference (CPP) [7,8]. Knockdown or over-expression of miR-212 levels in striatum is able to change cocaine intake behavior [9,10]. Deficiency of argonaute 2 (Ago2), a regulator site for miRNA-mediated gene silencing, in dopamine receptor 2-expressed neurons significantly reduces the motivation of cocaine intake in mice [11]. All these studies indicate that miRNAs may play important roles in the development of cocaine abuse. Notably, one miRNA is capable of regulating hundreds of mRNAs. On the other hand, mRNA level of a single gene might be controlled by different miRNAs [12]. Thus, miRNAs may be considered as “master regulators” or “bridge regulators” of gene expression.

Addiction-related abnormal memory is an important mechanism for compulsive cocaine-seeking and relapse behaviors [13]. Hippocampus, due to its key role in memory, has been suggested to be the key brain region involved in cocaine-seeking behavior [14]. Conditioned place preference (CPP) paradigm has been widely used to study the reward-related and drug contextual-associated memory of addictive drugs. For example, disruption of the hippocampal neural activity inhibits cocaine CPP [15], suggesting that hippocampus play an important role in cocaine CPP. Most of mechanisms related to drug addiction in hippocampus, such as synaptic modulation, glutamate receptors, neuronal plasticity, and dendritic morphology, likely involve miRNAs [16-19]. To date, studies on the roles of hippocampal miRNAs in various brain diseases have been targeted only to a small number of miRNAs. There is a lack of a broader view of cocaine-induced changes of miRNA expression within hippocampus.

In this study, we used miRNA microarray to examine changes in miRNA expression in rat hippocampus during the acquisition and extinction of cocaine-induced CPP. Potential targets of altered miRNA in this study were also discussed.

## Methods

### Animals

Adult male Wister rats (*n* = 68), weighing 250-280 g, were used in this study. Rats were maintained on a reverse light/dark cycle with food and water available *ad libitum*. All experiment procedures were approved by Animals Use and Care Committee of Nanjing Medical University, China. All efforts have been made to minimize both the suffering and the number of animals used.

### Drug treatment and conditioned place preference (CPP)

CPP was conducted in an apparatus constructed of three chambers (72 × 25 × 32 cm, Zhenghua Biologic Apparatus, Anhui, China). The two side larger chambers differ in their walls (black or black with white stripes, respectively) and floors (stainless-steel mesh or stainless-steel bars, respectively). The middle smaller chamber (11 × 25 × 32 cm) has gray wall with a smooth PVC floor. Three distinct chambers are separated by removable guillotine doors. Time spent in each chamber was recorded by means of infrared beam crossings which are located in the walls in each chamber.

**Figure 1:**
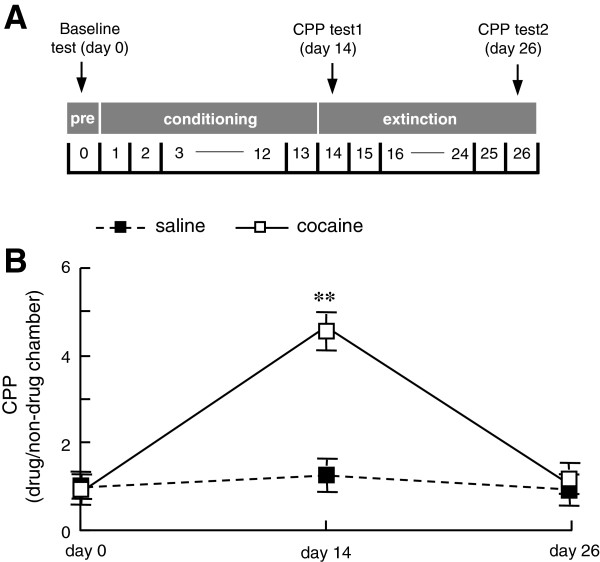
**CPP paradigm in this study. (A)** Timeline of CPP procedure. **(B)** Acquisition and extinction of cocaine-induced CPP. CPP score is the time spent in the drug-paired chamber versus in non-drug-paired chamber. Baseline is examined on day 0. CPP test 1 is performed on day 14 following 13-day CPP training and drug administration. CPP test 2 is carried out on day 26 after 12-day CPP extinction training. *n* = 12 ~ 16 rats per group. **p < 0.01 vs. baseline on day 0.

The place preference procedure consisted of three phases: pre-conditioning phase (baseline), conditioning (training), and post-conditioning test (CPP test). The detailed CPP timeline of this study were shown in Figure 1A. On day 0, the rats were free to explore the three chambers for 15 min. Rats that spent more than 500 s in one chamber were discarded. The CPP score is the ratio of the time spent in the drug-paired chamber to the time spent in non-drug-paired chamber. CPP training was performed for 13 consecutive days (day 1–13) with injections of cocaine hydrochloride (15 mg/kg, i.p., Qinghai Pharmaceutical, Qinghai, China) once daily. Controls were performed by injections of saline (0.5 ml/kg, i.p.) once daily. After each injection, the rats were confined to one conditioning side chamber (drug-paired chamber) for 45 min. CPP test 1 was carried out on day 14. In this phase, the rats freely moved throughout the apparatus for 15 min. CPP is considered to be acquired when CPP score is significantly higher in CPP test 1 than baseline. After the CPP test 1, rats were subjected to extinction phase of cocaine conditioning. During extinction period, all rats were confined to drug-paired chamber for 45 min each day without any drug injections. CPP is considered to be extinct when there is no significant difference between the CPP score in CPP test 2 and baseline. Rats acquired cocaine CPP on day 15 were defined as CCA group. Rats with saline CPP training served as SCA control. Rats that exhibited cocaine-extinct CPP were defined as CCE group. Rats with saline CPP and subsequent extinction trainings served as SCE control. *n* = 12 ~ 16 rats per group.

### RNA isolation

Rats were killed on day 14 after CPP test 1 and on day 26 after CPP test 2. The brains were immediately harvested on ice. Repeated cocaine-exposed rats that acquired cocaine CPP served as “CCA” group, and repeated saline-exposed rats served as SCA control. The cocaine CPP-extinguished rats served as CCE group, and rats with saline CPP and subsequent extinction trainings served as SCE control. Hippocampus was quickly removed from each brain on dry ice and stored at −80°C until use. To obtain enough RNA for detecting miRNA, hippocampus from 4 rats in one group were pooled. The following experiments were performed on RNA isolated from pooled samples (*n* = 3 samples/12 rats per group). Total RNA of hippocampus was extracted using TRIzol (Invitrogen, Carlsbad, CA, USA) and miRNasey mini Kit (Qiagen, Venlo, Netherlands) according to the manufacturer’s instructions. The quality and quantity of RNA were assessed by using NanoDrop-1000 UV–vis spectrophotometer (Midland, ON, Canada) and RNA integrity was determined by gel electrophoresis.

### MiRNA microarray

After RNA isolation from the samples, the miRCURY™ Hy3™/Hy5™ Power labeling kit (Exiqon, Vedbaek, Denmark) was used according to the manufacturer’s guideline for miRNA labelling. One microgram of each sample was 3′-end-labeled with Hy3™ fluorescent label, using T4 RNA ligase by the following procedure: RNA in 2.0 μL of water was combined with 1.0 μL of CIP buffer and CIP (Exiqon, Vedbaek, Denmark). The mixture was incubated for 30 min at 37°C, and was terminated by incubation for 5 min at 95°C. Then 3.0 μL of labeling buffer, 1.5 μL of fluorescent label (Hy3™), 2.0 μL of DMSO, 2.0 μL of labeling enzyme were added into the mixture. The labeling reaction was incubated for 1 h at 16°C, and terminated by incubation for 15 min at 65°C.

After stopping the labeling procedure, the Hy3™-labeled samples were hybridized on the miRCURY™ LNA Array (v.16.0) (Exiqon, Vedbaek, Denmark) according to array manual. The total 25 μL mixture from Hy3™-labeled samples with 25 μL hybridization buffer were first denatured for 2 min at 95°C, incubated on ice for 2 min and then hybridized to the microarray for 16 – 20 h at 56°C in a 12-Bay Hybridization Systems (Hybridization System - Nimblegen Systems, Inc., Madison, WI, USA), which provides an active mixing action and constant incubation temperature to improve hybridization uniformity and enhance signal. Following hybridization, the slides were achieved, washed several times using Wash buffer kit (Exiqon, Vedbaek, Denmark), and finally dried by centrifugation for 5 min at 400 rpm. Then the slides were scanned using the Axon GenePix 4000B microarray scanner (Axon Instruments, Foster City, CA, USA).

Scanned images were then imported into GenePix Pro 6.0 software (Axon Instruments, Foster City, CA, USA) for grid alignment and data extraction. The miRNAs, whose intensities are more than 50 in all samples, were chosen for calculating normalization factor. Expressed data were normalized using the Median normalization. After normalization, significant differentially expressed miRNAs were identified through Volcano Plot filtering. Hierarchical clustering was performed using MEV software (v4.6).

### MiRNA qRT-PCR

RNA samples that were applied to microarray were also used for qRT-PCR confirmation. Total RNA were reversely transcripted (RT) using mature miRNA-specific RT primers (Additional file 1), and subsequent qPCR reaction was performed using the SYBR Green PCR Master Mix Kit (SuperArray) and ABI 7900 fast real-time PCR system (Applied Biosystems). The 10-μl PCR reaction system contained: 5 μL 2 × master mix, 1 μL miRNA specific primer sets (Additional file 1), 2 μL miRNA RT product, and 2 μL ddH_2_O. Each reaction sample was run in triplicates. The PCR reactions were incubated at 95°C for 30 min, followed by 40 cycles of 95°C for 10 s, 60°C for 60 s. The expression of U6 small nucleolar RNA was used as internal control. The relative expression level for each miRNA was calculated by the comparative CT method (2^−ΔΔCt^).

### MiRNA target gene prediction and their functional analysis

The target genes of differentially expressed miRNA in this study were predicted using miRanda (http://www.microrna.org/), miRBase (http://mirbase.org/) and miRDB (http://mirdb.org/) databases. In order to eliminate false-positive predictions, only the overlapped target genes in the three databases were selected to perform the subsequent functional analysis. The function classification of predicted miRNA target genes, including molecular function, biological process and cellular component, were performed based on universal Gene Ontology (GO) terms (http://bioinfo.vanderbile.edu/webgestalt/). KEGG Mapper program (http://www.genome.jp/kegg/) was used here to investigate the functional pathways in which these predicted target genes were involved.

### Statistical analysis

For CPP data, CPP score is the ratio of the time spent in the drug-paired chamber to the time spent in non-drug-paired chamber, and data are expressed as mean ± SD. For miRNA array, data are expressed as normalized median. For qRT-PCR, data are expressed as mean ± SD. Differences among SCA, CCA, SCE and CCE groups were analyzed by ANOVA with Student-Newman-Keuls (*q* test). The significance is all set at *p* < 0.05, and fold change cut-off is 1.5 for microarray. Pearson correlation test is performed between the data of microarray study and that of qRT-PCR.

## Results

### MiRNA expression in hippocampus during acquisition and extinction of cocaine CPP in rats

At day 14 after receiving 13 days of cocaine administration and CPP training, rats (CCA group) spent more time in drug-paired chamber than in non-drug-paired chambers (*p* < 0.05 vs. baseline on day 0, Figure 1B), suggesting that CCA rats acquired cocaine-induced CPP behavior. In contrast, the saline-treated rats did not show place preference (*p* > 0.05 vs. baseline on day 0, Figure 1B), and hence are used as control (SCA). Expression of miRNA in rat hippocampus was examined using the sixth generation of miRCURY™ LNA Array (v.16.0, Exiqon). As shown in Table 1, 34 miRNA were significantly changed in hippocampal expressions in CCA rats, as compared to that in SCA rats (fold change > = 1.5, *p* < 0.05). Among them, 25 miRNAs levels were up-regulated, whereas 9 miRNAs levels were down-regulated following repeated cocaine exposure.

**Table 1:** Differentially expressed miRNAs in hippocampus when rats acquired cocaine CPP

**miRNA**	**SCA**	**CCA**	**Ratio of CCA to SCA**	**Regulation**^**a**^
miR-134	0.006139	0.039531	6.44	up
miR-152	0.01482	0.058302	3.93	up
miR-154*	0.015134	0.041161	2.72	up
miR-181c	0.070352	0.119279	1.70	up
miR-181c*	0.092892	0.269775	2.90	up
miR-194	0.032277	0.060462	1.87	up
miR-199a-3p	0.014452	0.075678	5.24	up
miR-26b	1.859555	2.820702	1.52	up
miR-30a*	0.006172	0.040441	6.55	up
miR-344b-2-3p	0.23734	0.396449	1.67	up
miR-34b	0.240389	0.43971	1.83	up
miR-34c	0.16784	0.307303	1.83	up
miR-350	0.149637	0.241594	1.61	up
miR-3597-5p	0.636307	0.988536	1.55	up
miR-369-5p	0.244867	0.368477	1.50	up
miR-409-3p	0.017127	0.054199	3.16	up
miR-423*	0.093672	0.171984	1.84	up
miR-431	0.048806	0.107651	2.21	up
miR-465*	0.021505	0.047859	2.23	up
miR-504	0.015596	0.058488	3.75	up
miR-540*	0.018673	0.051985	2.78	up
miR-542-3p	0.020206	0.059183	2.93	up
miR-665	0.065823	0.102721	1.56	up
miR-674-3p	0.057451	0.117307	2.04	up
miR-770	0.028775	0.047354	1.65	up
miR-872	0.079561	0.134929	1.70	up
miR-874	0.035205	0.058923	1.67	up
miR-877	0.024626	0.061976	2.52	up
miR-878	0.11178	0.181681	1.63	up
miR-133b	0.128301	0.044647	0.35	down
miR-144	0.326406	0.203167	0.62	down
miR-191	5.170566	2.72656	0.53	down
miR-224*	0.245938	0.006648	0.03	down
miR-451	1.690767	1.024982	0.61	down

Data are expressed as normalized median. SCA, repeated saline-exposed rats with CPP procedure; CCA, repeated cocaine-exposed rats that acquired cocaine CPP.

a, *p* < 0.05 vs. SCA, and fold change > = 1.5.

At day 26 following 12-day extinction training of cocaine treatment, rats with cocaine-treated history (CCE group) showed similar CPP score as measured in pre-conditioning phase (*p* > 0.05 vs. baseline on day 0), indicating that the cocaine-induced CPP has diminished (Figure 1B). Rats with saline CPP training and CPP extinction training were served as normal control (SCE group). Microarray study showed that 33 miRNAs levels were up-regulated and 9 miRNAs levels were down-regulated in hippocampus in CCE rats on day 26, as compared to that in SCE rats (fold changed > = 1.5, *p* < 0.05 vs. SCE control, Table 2).

**Table 2:** Differentially expressed miRNAs in hippocampus when cocaine-induced CPP were extinct in rats

**miRNA**	**SCE**	**CCE**	**Ratio of CCA to SCA**	**Regulation**^**a**^
miR-129	0.615865	1.03872	1.69	up
miR-134	0.006268	0.047953	7.65	up
miR-135a	1.209462	2.791499	2.31	up
miR-152	0.014801	0.058629	3.96	up
miR-154*	0.016234	0.055953	3.45	up
miR-181c	0.070112	0.157809	2.25	up
miR-181c*	0.102808	0.280031	2.72	up
miR-190	0.66164	1.05101	1.59	up
miR-194	0.031437	0.065415	2.08	up
miR-22	2.387086	3.989669	1.67	up
miR-26b	1.857727	3.041154	1.64	up
miR-30a*	0.006116	0.041296	6.75	up
miR-350	0.148751	0.238601	1.60	up
miR-3597-5p	0.635073	1.198465	1.89	up
miR-369-5p	0.246427	0.442758	1.80	up
miR-376c*	0.166055	0.327697	1.97	up
miR-380*	0.151079	0.278278	1.84	up
miR-382	0.444579	0.732439	1.65	up
miR-383	0.06203	0.1888	3.04	up
miR-409-3p	0.01797	0.049711	2.77	up
miR-423*	0.093596	0.149149	1.59	up
miR-431	0.04564	0.089228	1.96	up
miR-540*	0.01814	0.050267	2.77	up
miR-542-3p	0.02204	0.048215	2.19	up
miR-665	0.060955	0.094727	1.55	up
miR-674-3p	0.059617	0.139871	2.35	up
miR-702-3p	0.058183	0.089527	1.54	up
miR-708	0.460416	0.86281	1.87	up
miR-770	0.029214	0.078812	2.70	up
miR-872	0.081533	0.129905	1.59	up
miR-873	0.13028	0.244599	1.88	up
miR-874	0.036179	0.058688	1.62	up
miR-878	0.113067	0.175961	1.56	up
miR-133b	0.143561	0.044991	0.31	down
miR-144	0.339555	0.102345	0.30	down
miR-191	5.370077	2.882197	0.54	down
miR-224*	0.255883	0.008133	0.03	down
miR-347	0.264399	0.173543	0.66	down
miR-34b	0.244946	0.143185	0.58	down
miR-34c	0.167814	0.094695	0.56	down
miR-451	1.294431	0.448675	0.35	down
miR-99b*	0.079249	0.051688	0.65	down

Data are expressed as normalized median. SCE, rats with CPP procedure and subsequent extinction trainings; CCE, cocaine CPP-extinguished rats.

a, *p* < 0.05 vs. SCE, and fold change > = 1.5.

### Validation of miRNA array findings by qRT-PCR

**Figure 2:**
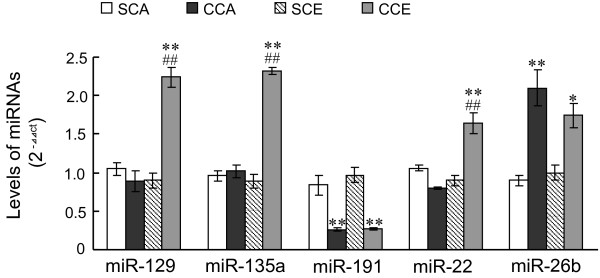
**Levels of five miRNAs in rat hippocampus by qRT-PCR.** Values are mean ± SD. Repeated cocaine-exposed rats that acquired cocaine CPP serves as “CCA” group, and repeated saline-exposed rats serves as SCA control. The cocaine CPP-extinguished rats serves as CCE group, and rats with saline CPP and subsequent extinction trainings serves as SCE control. *n* = 3 samples/12 rats per group. **p < 0.01, *p < 0.05 vs. controls (SCA or SCE rats). ##*p* < 0.01 vs. CCA rats.

To confirm findings from miRNA array study, five miRNAs (miR-129, miR-135a, miR-191, miRNA-22 and miR-26b) were chosen to examine their expression in rat hippocampus by quantitative RT-PCR (qRT-PCR). As shown in Figure 2, levels of miR-129, miR-135a and miRNA-22 were significantly up-regulated in hippocampus in CCE rats, as compared to that in SCE rats (*p* < 0.05). Repeated cocaine exposure produced an increase in expression of miR-26b and a decrease in expression of miR-191 in CCA rats (*p* < 0.05 vs. SCA), and these changes were still evident in CCE rats (*p* < 0.05 vs. SCA). There is a strong correlation between data from array study and qRT-PCR study (*r*^*2*^ = 0.95772).

### Comparison of miRNA expression in hippocampus between CCA and CCE rats

**Figure 3:**
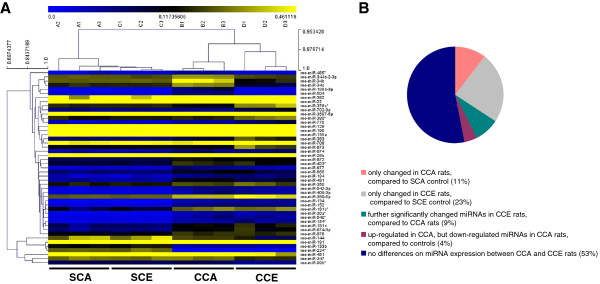
**Comparison of miRNA expression in hippocampus between CCA and CCE rats. (A)** Hierarchial-clustering map. Rows, individual miRNA; columns, individual sample. A1-3, B1-3, C1-3 and D1-3 are the sample numbers for each group. Values are normalized median. *n* = 3 samples/12 rats per group. **(B)** Comparison of the miRNA expression patterns between CCA and CCE rats. Altered hippocampal miRNAs in this study are divided into 5 classes according to their expression patterns in CCA and CCE rats. The percentage of each class was shown in the pie-chart.

To compare the expression of hippocampal miRNAs between CCA and CCE rats, a hierarchial clustering map of these miRNAs was created (Figure 3A). As shown in Figure 3B and Additional file 2, levels of miR-199a-3p, miR-344b-2-3p, miR-465*, miR-504, and miR-877 showed selectively changed in CCA rats (*p* < 0.05 vs. SCA), but not altered in CCE rats (*p* > 0.05 vs. SCE). Twelve miRNAs (miR-135a, miR-190, miR-22, miR-347, miR-376*, miR-380*, miR-382, miR-383, miR-702-3p, miR-708, miR-873, and miR-99b*) were regulated only in CCE rats (*p* < 0.05 vs. SCE), but failed to be altered in CCA rats (*p* > 0.05 vs. SCA). Levels of miR-144, miR-451, and miR-770 were significantly down-regulated in CCA rats (*p* < 0.05 vs. SCA), and got further decrease in CCE rats (*p* < 0.05 vs. SCE, and *p* < 0.05 vs. CCA). Levels of miR-34b and miR-34c, which showed an increase in expression in CCA rats (*p* < 0.05 vs. SCA), were significantly down-regulated in CCE rats (*p* < 0.05 vs. SCE, and *p* < 0.05 vs. CCA).

### MicroRNA target gene prediction and function analysis

**Figure 4:**
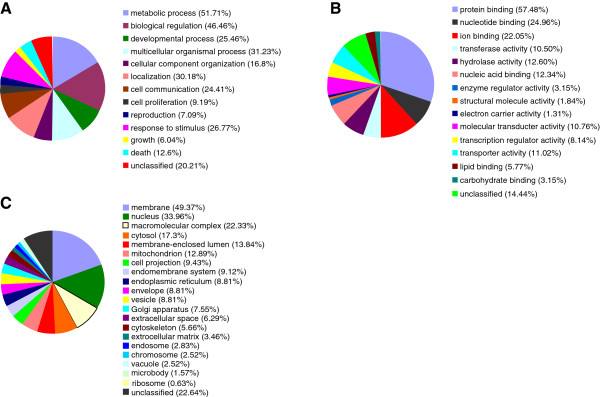
**GO analysis of the predicted miRNA targets.** The percentage of each class was shown in the pie-chart. **(A)** Distribution of biological process. **(B)** Distribution of molecular function. **(C)** Distribution of cellular component.

The potential targets of the altered miRNAs identified in CCA and CCE rats were predicted and integrated by three micoRNA target prediction databases (miRanda, miRBase and miRDB). In total, 403 targets were identified. To understand the functional implications of these target genes, the predicted target genes were put into GO analysis. The 403 targets were classed into 13 sorts according to biological process (Figure 4), most of which are involved in metabolic process (51.71%) and biological regulation (46.46%). As to molecular function, the analysis generated 15 function classes. Many of these targets are involved in protein binding (57.48%) and nucleotide binding (24.96%). For cellular component analysis, these targets are mostly located at membrane (49.37%) and nucleus (33.96%). KEGG pathway analysis on miRNA target genes was also performed. We focus on analyzing the miRNAs and their potential target genes that may be involved in nervous system-related pathways and signal transduction pathways (Table 3).

**Table 3:** KEGG analysis of predicted target genes of miRNAs in rat hippocampus

**Pathway term**	**miRNA target (gene_symbol)**	**miRNA**
Glutamatergic Synapse	Gnai3, Gng10, Pla2g4a, Plcb4, Slc17a6, Chp2	miR-135a, miR-144, miR-22, miR-350, miR-770
Gabaergic Synapse	Gnai3, Gng10, Plcl1, Slc38a5	miR-144, miR-22, miR-34c, miR-674-3p, miR-873, miR-878
Cholinergic Synapse	Chrm3, Gnai3, Gng10, Plcb4, Bcl2	miR-22, miR-144, miR-770, miR-34b, miR-34c, miR-878
Dopaminergic Synapse	Gnai3, Gng10, Kif5a, Arrb2, Plcb4, Ppp2cb	miR-22, miR-144, miR-347, miR-181c, miR-30a*, miR-770, miR-26b, miR-133b, miR-878
Serotonergic Synapse	Gnai3, Gng10, Pla2g4a, Plcb4, Ptgs1, Raf1	miR-22, miR-144, miR-770, miR-181c, miR-382, miR-873, miR-770
Long-Term Potentiation	Plcb4, Raf1, Chp2	miR-770, miR-135a, miR-350
Long-Term Depression	Gnai3, Pla2g4a, Plcb4, Ppp2cb, Raf1	miR-22, miR-144, miR-770, miR-26b, miR-133b, miR-878
Retrograde Endocannabinoid Signaling	Gnai3, Gng10, Plcb4, Slc17a6	miR-22, miR-144, miR-770, miR-350
Synaptic Vesicle Cycle	Cplx1, Clta, Cltc, Ap2a2, Atp6v1e1, Slc17a6, Vamp2, Atp6v0d1	miR-129, miR-135a, miR-133b, miR-350, miR-152, miR-34c, miR-194, miR-431, miR-382, miR-872
Neurotrophin Signaling Pathway	Prkcd, Raf1, Bcl2, Tp53	miR-181c, miR-26b, miR-350, miR-34c, miR-874, miR-34b, miR-770
Alzheimer’s Disease	Atp5g3, Plcb4, Sdhd, Chp2, Adam17, Uqcrc2	miR-34b, miR-878, miR-770, miR-674-3p, miR-135a, miR-350, miR-194, miR-26b
Cocaine Addiction	Gnai3	miR-22
Amphetamine Addiction	Chp2	miR-135a, miR-350
MAPK Signaling Pathway	Gadd45a, Arrb2, Pak1, Pla2g4a, Raf1, Chp2, Stk3, Tp53, Map3k12, Cacna2d1	miR-134, miR-152, miR-431, miR-181c, miR-30a*, miR-144, miR-770, miR-135a, miR-350, miR-665, miR-34b, miR-34c, miR-874, miR-191
Calcium Signaling Pathway	Chrm3, Adrb3, Slc25a5, Pde1c, Phkg2, Plcb4, Plcd1, Ptafr, Chp2, Cckbr	miR-878, miR-26b, miR-347, miR-134, miR-135a, miR-350, miR-674-3p, miR-770, miR-191, miR-152, miR-30a*
PI3K-Akt signaling pathway	Cdk2, Col2a1, Col4a4, Csf1r, Gng10, Hgf, Pck1, Ppp2cb, Ptk2, Raf1, Bcl2, Tlr2, Tp53, Ywhag	miR-134, miR-152, miR-26b, miR-133b, miR-878, miR-34b, miR-34c, miR-872, miR-144, miR-135a, miR-770, miR-181c

## Discussion

The regulation of gene expression in hippocampus is thought to contribute to the development of drug addiction [13,14]. The miRNAs, the key regulators for of gene expression, are emerging as vital regulatory components of drug addiction [7-11]. In this study, 47 miRNAs were found to be regulated in rat hippocampus in response to cocaine exposure and subsequent cocaine absence.

Our result indicates that miRNA expression profiles of hippocampus are different in cocaine CPP-acquiring phase and CPP-extinguishing phase. Repeated cocaine exposure may produce changes in miRNA expression, which may lead to alteration in gene expression, and result in structural and functional modifications in hippocampus. Ultimately, these modifications might contribute to drug addictive behaviors, such as cocaine CPP. Some alteration in miRNA expression following cocaine exposure may persist for a long time, even still being evident after a long-term abstinence from cocaine administration, such as miR-144, miR-451 and miR-770 in this study. We hypothesized that long-lasting alterations in miRNA expression may contribute to persistent memory of drug-related euphoric events and contexts, and ultimately result in cocaine relapse. Notably, some miRNAs levels selectively affected in CCE rats, but not in CCA rats, such as miR-129, miR-135a and miR-22 in this study. In addition, levels of miR-34b and miR-34c were up-regulated in CCA rats, but down-regulated in CCE rats. These findings indicate that abstinence from previous cocaine experiences also has impacts on miRNA expression in hippocampus. Further studies are needed to investigate the contributions of these cocaine treatment-induced dynamic alterations of miRNA expression in rat hippocampus to drug-associated memory in the brain.

So far, little is known about the correlation between hippocampal miRNA and drug abuse. Several miRNAs altered following cocaine treatment in this study were suggested to be important regulators in hippocampus. For example, miR-134 is a brain-specific miRNA that can negatively regulate the size of dendritic spines of rat hippocampal neurons via mediating limk1 expression [19]. Silencing miR-134 expression in hippocampus *in vivo* reduces the prolonged seizures in mice [20]. Other miRNAs, such as miR-181c, miR-191, miR-22, miR-99b*, and miR-369-5p, are also differentially modulated in rat hippocampus during post-status epilepsy [21,22]. The level of miR-34c is found to be elevated in the hippocampus in Alzheimer’s disease [23]. These results suggest that these miRNAs may be involved in some pathological disorders occurred in hippocampus, but whether they exert regulatory effects on cocaine abuse remains to be investigated.

In order to select candidate hippocampal miRNAs that may be important to cocaine abuse, we compared the current findings with that of previous studies. Several miRNAs indentified in this study showed to be involved in the addiction process of cocaine and other abused drugs. For instance, chronic cocaine exposure also changes the levels of miR-382 and miR-409-3p in nucleus accumbens [12]. The level of miR-133b is regulated also by morphine in hippocampal neurons [24], and pregnant cocaine exposure is able to affect the expression of dopamine receptors (DR) in embryos via miR-133b regulation [25]. Other examples include miR-504 and miR-26b. MiR-504 mediates expression of the dopamine D1 receptor [26] and miR-26b is reported to negatively regulate brain-derived neurotrophic factor (BDNF) expression at post-transcriptional level [27]. DR and BDNF are key molecules involved in cocaine addiction [28,29]. These comparisons indicate that these miRNAs may be vulnerable in nervous disorders and may be common targets of drug abuse. It is of great interest to study the roles of these miRNAs in the development of cognitive deficits associated with drug abuse in future. Recently, some miRNAs in the nucleus accumbens have been reported to be involved in behavioral changes in cocaine CPP, such as miR-181a, miR-124 and let-7b [7,8]. MiR-212 in the striatum is able to regulate cocaine self-administrated intake behaviors [9,10]. In this study, no changes had been detected in their expressions in rat hippocampus following acquisition and extinction of cocaine CPP. On one hand, miRNA may perform their mediating roles in cocaine abuse in a brain region-specific manner. On the other hand, the levels of miRNAs in the brain may be changed depending on different abuse animal models, such as different drug doses, different drug timelines, self-administration models and CPP models.

To gain further insight into the roles of miRNAs in cocaine abuse, the potential gene targets of differentially expressed hippocampal miRNAs were predicted. To better understand the possible functions of these target genes, GO analysis and KEEG pathway analysis were performed. Cocaine abuse has been thought to influence brain activity through modulating signaling pathways and synaptic function in the brain [2,3]. Hence, we paid more attention to the miRNAs targets that may be involved in nervous system-related pathways and signal translating pathways (as shown in Table 3). There were several target genes that attracted our attention. The first example is Raf1. miR-212 in striatum is reported to decrease responsiveness to the motivational properties of cocaine by activating CREB signaling. This process requires the enhancement of Raf1 activities [9]. miR-770, which was up-regulated in this study, may exert regulatory actions on the activity of Raf1. The other example is Gnai3, a G-protein alpha3 inhibiting activity polypeptide. Gnai3 protein together with beta-gamma dimmers and an activator of G-protein signaling 3 may mediate the relapse of herion-seeking behavior [30]. In addition, many G-protein-coupled receptors, such as 5-hydroxytryptamine-A1 receptor, GABA_B_ receptor and cannabinoid CB1 receptor, mediate synaptic transmission in hippocampal neurons by binding to Gnai3 protein [31,32]. In this study, several altered hippocampal miRNA, including miR-133b, miR-152, miR-194, miR-22, miR-30a*, miR-347, and miR-874, have potential ability to regulate the expression of Gnai3.

## Conclusions

To our knowledge, this is the first study to reveal different miRNAs expression patterns in rat hippocampus during the acquisition and extinction of cocaine-induced CPP. These findings may extend our understanding of the regulatory network underlying cocaine abuse and may provide new targets for the future treatment of drug abuse. Notably, we only explored the global miRNA changes in rat hippocampus using cocaine CPP model. Future studies are needed to characterize the physiological implications of these altered miRNAs in drug of abuse.

## Competing interests

The authors declare that they have no competing interests.

## Authors’ contributions

CLC and HL participated in collection of samples, RNA isolation, behavioral tests and real time RT-PCR experiments. XG contributed to the animal surgery, the concept and design of the study, the data analysis and interpretation, and the writing of the manuscript. All authors read and approved the final manuscript.

## Supplementary Material

Additional file 1 Specific RT primers and PCR primers. Click here for file

Additional file 2 **Differences in hippocampal miRNA expressions between CCA and CCE rats.***Description:* All miRNAs presented in this table show significant difference in their expressions between CCA and CCE rats, *p* < 0.05 vs. CCE. up, up-regulated; down, down-regulated; n, no change. Regulation1, miRNAs levels are regulated in CCA rats, as compared to SCA control (fold change > = 1.5, *p* < 0.05). Regulation2, miRNAs levels are regulated in CCE rats, as compared to SCE control (fold change > = 1.5, *p* < 0.05). Click here for file
