# Supplementary material for: Changes in microRNA expression profile in hippocampus during the acquisition and extinction of cocaine-induced conditioned place preference in rats
Source: J Biomed Sci. 2013 Dec 20;20(1):96. doi: 10.1186/1423-0127-20-96 (PMC3878172; doi:10.1186/1423-0127-20-96)
Supplement: Additional file 1 — Specific RT primers and PCR primers. [file 1423-0127-20-96-S1.doc]

**Additional File 1** Specific RT primers and PCR primers

| Gene name | RT prime | PCR primers |
| --- | --- | --- |
| U6 | 5’CGCTTCACGAATTTGCGTGTCAT3’ | F: 5'GCTTCGGCAGCACATATACTAAAAT3' |
|  |  | R:5'CGCTTCACGAATTTGCGTGTCAT3' |
| rno-miR-129 | 5’GTCGTATCCAGTGCGTGTCGTGGAGTCGGCAATTGCACTGGATACGACGCAAGC3’ | GSP:5'GCTTTTTGCGGTCTGG3' |
|  |  | R:5'TGCGTGTCGTGGAGTC3' |
| rno-miR-135a | 5’GTCGTATCCAGTGCGTGTCGTGGAGTCGGCAATTGCACTGGATACGACTCACAT3’ | GSP:5'GGGTATGGCTTTTTATTCCT3' |
|  |  | R:5'CAGTGCGTGTCGTGGAGT3' |
| rno-miR-191 | 5’GTCGTATCCAGTGCGTGTCGTGGAGTCGGCAATTGCACTGGATACGACCAGCTG3’ | GSP:5'CAACGGAATCCCAAAAG3' |
|  |  | R:5'CAGTGCGTGTCGTGGA3' |
| rno-miR-22 | 5’GTCGTATCCAGTGCGTGTCGTGGAGTCGGCAATTGCACTGGATACGACACAGTT3’ | GSP:5'GGAAGCTGCCAGTTGAAG3' |
|  |  | R:5'CAGTGCGTGTCGTGGAGT3' |
| rno-miR-26b | 5’GTCGTATCCAGTGCGTGTCGTGGAGTCGGCAATTGCACTGGATACGACACCTAT3’ | GSP:5'GGGGTTCAAGTAATTCAGG3' |
|  |  | R:5'CAGTGCGTGTCGTGGA3' |
