# Supplementary material for: Changes in microRNA expression profile in hippocampus during the acquisition and extinction of cocaine-induced conditioned place preference in rats
Source: J Biomed Sci. 2013 Dec 20;20(1):96. doi: 10.1186/1423-0127-20-96 (PMC3878172; doi:10.1186/1423-0127-20-96)
Supplement: Additional file 2 — Differences in hippocampal miRNA expressions between CCA and CCE rats.Description: All miRNAs presented in this table show significant difference in their expressions between CCA and CCE rats, p < 0.05 vs. CCE. up, up-regulated; down, down-regulated; n, no change. Regulation1, miRNAs levels are regulated in CCA rats, as compared to SCA control (fold change > = 1.5, p < 0.05). Regulation2, miRNAs levels are regulated in CCE rats, as compared to SCE control (fold change > = 1.5, p < 0.05). [file 1423-0127-20-96-S2.doc]

**Additional file 2** Differences in hippocampal miRNA expressions between CCA and CCE rats

| MiRNA | Regulation1 | Regulation2 |
| --- | --- | --- |
| miR-129 | n | up |
| miR-135a | n | up |
| miR-144 | down | down |
| miR-190 | n | up |
| miR-199a-3p | up | n |
| miR-22 | n | up |
| miR-344b-2-3p | up | n |
| miR-347 | n | down |
| miR-34b | up | down |
| miR-34c | up | down |
| miR-376c* | n | up |
| miR-380* | n | up |
| miR-382 | n | up |
| miR-383 | n | up |
| miR-451 | down | down |
| miR-465* | up | n |
| miR-504 | up | n |
| miR-702-3p | n | up |
| miR-708 | N | up |
| miR-770 | up | up |
| miR-873 | n | up |
| miR-877 | up | n |
| miR-99b* | n | down |

All miRNAs presented in this table show significant difference in their expressions between CCA and CCE rats, *p* < 0.05 vs. CCE

up, up-regulated; down, down-regulated; n, no change.

Regulation1, miRNAs levels are regulated in CCA rats, as compared to SCA control (fold change > = 1.5, *p* < 0.05).

Regulation2, miRNAs levels are regulated in CCE rats, as compared to SCE control (fold change > = 1.5, *p* < 0.05).
